# Mediation Analysis using Semi-parametric Shape-Restricted Regression with Applications

**DOI:** 10.1007/s13571-024-00336-w

**Published:** 2024-07-02

**Authors:** Qing Yin, Jong-Hyeon Jeong, Xu Qin, Shyamal D Peddada, Jennifer J Adibi

**Affiliations:** 1 Department of Biostatistics, School of Public Health, University of Pittsburgh, Pittsburgh, PA USA; 2 Present Address: Division of Cancer Treatment and Diagnosis, National Cancer Institute, Bethesda, USA; 3 Department of Health and Human Development, School of Education, University of Pittsburgh, Pittsburgh, PA USA; 4 Biostatistics and Computational Biology Branch, National Institute of Environmental Health Sciences (NIEHS), NIH, Durham, NC USA; 5 Department of Epidemiology, School of Public Health, University of Pittsburgh, Pittsburgh, PA USA

**Keywords:** Birth-weight, Constrained inference, Human chorionic gonadotropin (hCG), Mediation analysis, Placental-fetal hormones, Pesticides exposure, Regression spline, Shape-restricted inference, Primary: 62D20, Secondary: 62F30

## Abstract

Often linear regression is used to estimate mediation effects. In many instances the underlying relationships may not be linear. Although, the exact functional form of the relationship may be unknown, based on the underlying science, one may hypothesize the shape of the relationship. For these reasons, we develop a novel shape-restricted inference-based methodology for conducting mediation analysis. This work is motivated by an application in fetal endocrinology where researchers are interested in understanding the effects of pesticide application on birth weight, with human chorionic gonadotropin (hCG) as the mediator. Using the proposed methodology on a population-level prenatal screening program data, with hCG as the mediator, we discovered that while the natural direct effects suggest a positive association between pesticide application and birth weight, the natural indirect effects were negative.

**Supplementary Information:**

The online version contains supplementary material available at 10.1007/s13571-024-00336-w.

## Introduction

Often in experimental sciences, such as in lab sciences, industrial quality control, agricultural sciences, one can conduct well-controlled studies to infer effects of a treatment. However, often in observational studies, such as in epidemiological studies, causal effects of a treatment or an exposure on the outcome cannot be ascertained using standard statistical methods. One can only infer associations. Over the past two decades, there has been considerable interest in developing statistical methods and framework to infer causality and mediation effects under some assumptions regarding the underlying models (VanderWeele 2015; Baron and Kenny 1986). Most statistical methods assume that the association of the expected value of the outcome variable with the mediator and other covariates is linear in the unknown parameters.

Linear regression, where the unknown parameters are modeled linearly, has a long history with well-developed theory and methodology (Rao 1973). However, nonlinear relationships are more commonly encountered in biomedical research than acknowledged or modeled (Sugihara et al. 2012). For instance, toxicologists often model dose-response relationships using the Hill function (Lim et al. 2013). Similar to linear models, theory and methodology for parametric nonlinear regression are well developed (Seber and Wild 1989; Davidian and Giltinan 1995).

However, there are many instances where the relationship between two variables is expected to be nonlinear but the exact functional forms are unknown. For example, in large scale time course omics studies involving thousands of features, such as gene expression, CpG site methylation, or microbiome, etc., different features may display different nonlinear time course patterns which are a priori unknown (Peddada et al. 2003). Similarly, in quantitative high throughput screening (qHTS) assays involving several thousands of chemicals conducted by agencies such as the US National Toxicology Program (NTP), the US Environmental Protection Agency (EPA), and others, the dose-response patterns for active compounds are not always linear, or even monotonic (Shockley et al. 2019). Due to the dose range considered, at some unknown high dose, some compounds may even change the direction of response, resulting in either a convex or concave shaped response. The proposed method is designed specifically to address non-linearity in the mediator-outcome relationship. This reflects the underlying endocrinology of placental-fetal development (Adibi et al. 2024).

**Fig. 1:**
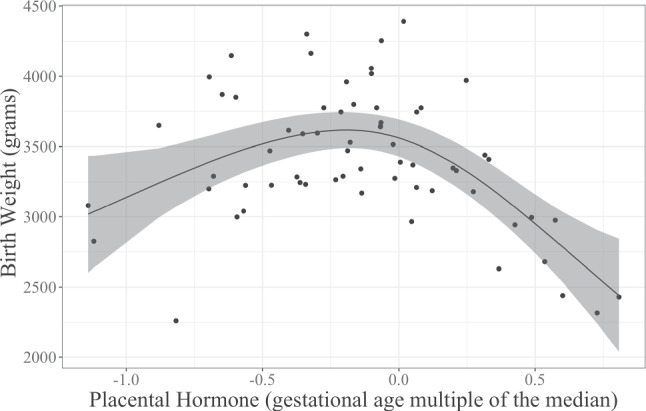
The relationship between a placental hormone (expressed as a gestational age multiple of median) and infant birth weight

Model misspecification may introduce bias in estimates and potentially lead to incorrect or even invalid conclusions and interpretations of the data. To deal with nonlinearity and unknown functional relationships, flexible Generalized Additive Models (GAMs) were considered by Imai et al. (2010) for performing mediation analysis, where the direct and indirect effects are estimated using simulations. While GAMs offer flexibility to model relationships between variables, they may result in arbitrary shapes for the mean response that are not scientifically plausible or easy to explain. In many scientific problems, the patterns of responses, though nonlinear, are not arbitrarily shaped but are determined by a biologic mechanism. The study design or scientific question may limit the patterns of response to a particular subset of shapes. For instance, the pattern of expression of a cell division cycle or a circadian clock gene is expected to be rhythmic over time, not necessarily sinusoidal (Larriba et al. 2016). In all such instances, it may be more appropriate to use semi-parametric shape restricted models where the expected responses are restricted to a particular class of shapes. Motivated by dose-response studies, such as those in toxicology and the fetal endocrinology described in this paper, we consider monotonic, convex or concave relationships between the expected value of the outcome variable and the mediator. An example of such a nonlinear relationship is shown in Fig. 1, where the relationship between placental hormone and infant birth weight is concave.

The paper is organized as follows. Using semi-parametric shape-restricted regression splines (Meyer 2008, 2018; Yin et al. 2021), we develop outcome and mediator models in Section 2. The estimated parameters from these models are then used to estimate the direct and indirect effects of various factors and their confidence intervals. Using simulation studies where the models and parameters are inspired by our fetal endocrinology data, we investigate the performance of the estimators in Section 3. In Section 4, the proposed methodology is applied to a population-level prenatal screening program data set to describe the role of placental hormones as mediators in the relationship between pesticide application and the birth weight of infants. Concluding remarks are provided in Section 5. All mathematical details are provided in the supplementary text.

## Methodology

### Background and Notations

The regression-based mediation analysis is formulated within the “counterfactual” or sometimes called “potential outcomes” framework for causal inference. Within the “potential outcomes” framework, to obtain an estimable quantity, we require two rules to be satisfied: (i) consistency, and (ii) there is no unmeasured confounding (Pearl 2010; VanderWeele 2015). Note that the meaning of the term “consistency” in the causal inference literature is different from its meaning in traditional statistics literature, which refers to asymptotic properties of a statistical procedure. Before we describe these terms, we first provide some notations. For an individual *i*, let $A_i$ denote the exposure variable, $C_i$ denote the collection of confounding variables, and $Y_i$ denote the outcome variable. Let $Y_{i, A_i =a}$ denote the individual *i*’s potential outcome if the individual *i*’s exposure $A_i$ were set to a. Suppose, for the $i^{th}$ subject, $A_i = a$, then according to the consistency rule, $Y_{i, A_i = a} = Y_{i}$. In other words, the consistency rule states that for an individual *i* with actual exposure $A_i = a$, the actual outcome $Y_i$ is equal to $Y_{i, a}$. Alternatively, the consistency assumption states that $A_i$ is a well-defined intervention on the exposure that will consistently produce the same counterfactual outcome. For simplicity of notation, throughout the following we suppress the index *i*. Thus, under the consistency rule we have: $P(Y_a = y | C = c, A = a) = P(Y = y | C = c, A = a).$ The no unmeasured confounding rule states that given all confounding variables *C*, the potential outcome $Y_a$ is independent of the exposure *A*, i.e., $Y_a \perp (independent) A | C$. If the exposure variable *A* is binary, taking values $A = 0$ or $A = 1$, then under the above two rules, the average causal effect for a population given all confounding variables, $E[Y_1 - Y_0|C]$, can be identified and given by $E[Y|A = 1, C] - E[Y|A = 0, C]$, which can be estimated using the observed data. Let *M* denote the mediator variable, $M_a$ denote an individual’s potential mediator if the individual’s exposure *A* were set to *a*, and $Y_{am}$ denote an individual’s potential outcome if the individual’s exposure *A* were set to *a* and mediator *M* were set to *m*. The controlled direct effect (CDE), the natural direct effect (NDE) and the natural indirect effect (NIE) for a random subject are denoted by $Y_{am}-Y_{a^*m}$, $Y_{aM_{a^*}}-Y_{a^*M_{a^*}}$ and $Y_{aM_a}-Y_{aM_{a^*}}$, respectively. Note that $A = a$ and $A = a^*$ are two different exposure levels. For example, if the exposure is binary, then $a = 1$ and $a^* = 0$. For the identifiability of the CDE, we assume $Y_{am} \perp A | C$, which can be interpreted as conditional on C, there is no unmeasured exposure-outcome confounding, and $Y_{am} \perp M | {A, C}$, which can be interpreted as conditional on C, there is no unmeasured mediator-outcome confounding. For the identifiability of the NDE and NIE, besides the two assumptions above, we additionally assume $M_a \perp A | C$, which can be interpreted as conditional on C, there is no unmeasured exposure-mediator confounding, and $Y_{am} \perp M_{a^*} | C$, which can be interpreted as conditional on C, there is no mediator-outcome confounding affected by exposure.

For a real number *x*, and a sequence of knots $t = \{t_1, t_2, \ldots , t_{n+k}\}$, $M_i(x|k,t)$ denotes the $k^{th}$ order M-spline basis function, which is a piece-wise polynomial of degree $k - 1$ (Curry and Schoenberg 1966). The corresponding I-spline (Ramsay 1988) and C-spline (Meyer 2018) basis functions are given by $I_i(x|k,t) = \int _L^x M_i(u|k,t)du$ and $C_i(x|k,t) = \int _L^x I_i(u|k,t)du$, respectively.

The methodology developed in this paper relies on the general framework introduced in Meyer (2018) and Yin et al. (2021), which uses quadratic I-splines and cubic C-splines (see supplementary text for details). The quadratic I-splines are of interest in shape-restricted regression because a linear combination of quadratic I-spline basis functions is non-decreasing if and only if the coefficients are non-negative. The regression function using I-splines is estimated by a linear combination of the basis functions, the constant function, and other covariates (Meyer 2018). The cubic C-splines are of interest in shape-restricted regression because a linear combination of cubic C-spline basis functions is convex if and only if the coefficients are non-negative. The regression function using C-splines is estimated by a linear combination of the basis functions, the constant function, the identity function, and other covariates (Meyer 2018).

### Model

Suppose that the exposure is binary, i.e., an individual can be either exposed or non-exposed, an interaction between exposure and mediator exists, and the relationships between mediator and outcome in both exposure and non-exposure groups are potentially nonlinear. Then the exposure-outcome relationship is modeled by

1 $$\begin{aligned} Y = \beta _0 + \beta _1 A + f_1(M) A + f_2(M)(1 - A) + \beta _4 C + \epsilon _1, \end{aligned}$$

where $f_1(M)$ is the function of the mediator for the exposure group, $f_2(M)$ is the function of the mediator for the non-exposure group, and $\epsilon _1 \sim N(0, \sigma _1^2)$, and the exposure-mediator model is

2 $$\begin{aligned} M = \gamma _0 + \gamma _1 A + \gamma _2 C + \epsilon _2, \end{aligned}$$

where $$\epsilon _2 \sim N(0, \sigma _2^2)$$.

In the case of quadratic I-splines, $f_1(M) = \sum _{i=1}^k \beta _{2i} I_i(M|2, t)$ and $f_2(M) = \sum _{i=1}^k$ $\beta _{3i} I_i(M|2, t)$, and in case of cubic C-splines, $f_1(M) = \beta _{20}M + \sum _{i=1}^k \beta _{2i} C_i(M|2, t)$ and $f_2(M) = \beta _{30}M + \sum _{i=1}^k \beta _{3i} C_i(M|2, t)$, reflecting the structures of the I- and C-spline basis functions in the supplementary text. Define $IS = [I_1(M|2, t), ..., I_k(M|2, t)]$ and $CS = [M, C_1(M|2, t), ..., C_k(M|2, t)]$. Denote the symbol “$\bullet$” for the face-splitting product (for *A* = $$\begin{bmatrix} a_1 \\ a_2 \end{bmatrix}$$ and *B* = $$\begin{bmatrix} b_{11} &{} b_{12} \\ b_{21} &{} b_{22} \end{bmatrix}$$, the face-splitting product operates as $A \bullet B$ = $$\begin{bmatrix} a_1 b_{11} &{} a_1 b_{12} \\ a_2 b_{21} &{} a_2 b_{22} \end{bmatrix}$$), and let $\beta _2 = [\beta _{21}, ..., \beta _{2k}]$ if the corresponding matrix is $IS \bullet A$, or $\beta _2 = [\beta _{20}, \beta _{21}, ..., \beta _{2k}]$ if the corresponding matrix is $CS \bullet A$, and $\beta _3 = [\beta _{31}, ..., \beta _{3k}]$ if the corresponding matrix is $IS \bullet (1 - A)$, or $\beta _3 = [\beta _{30}, \beta _{31}, ..., \beta _{3k}]$ if the corresponding matrix is $CS \bullet (1 - A)$.

If $f_1(M)$ and $f_2(M)$ are both monotonic, then they are fitted using the I-splines, and model (1) is given by

3 $$\begin{aligned} \begin{aligned} Y&= [1, A, IS \bullet A, IS \bullet (1 - A), C] [\beta _0, \beta _1, \beta _{2}, \beta _{3}, \beta _4]^T + \epsilon _1. \end{aligned} \end{aligned}$$

If $f_1(M)$ and $f_2(M)$ are both convex (or concave), then they are fitted using the C-splines, and the model (1) is given by

4 $$\begin{aligned} \begin{aligned} Y&= [1, A, CS \bullet A, CS \bullet (1 - A), C] [\beta _0, \beta _1, \beta _{2}, \beta _{3}, \beta _4]^T + \epsilon _1. \end{aligned} \end{aligned}$$

If $f_1(M)$ is monotonic but $f_2(M)$ is convex (or concave), then $f_1(M)$ is fitted using I-splines and $f_2(M)$ is fitted using C-splines, and the model (1) is given by

5 $$\begin{aligned} \begin{aligned} Y&= [1, A, IS \bullet A, CS \bullet (1 - A), C] [\beta _0, \beta _1, \beta _{2}, \beta _{3}, \beta _4]^T + \epsilon _1. \end{aligned} \end{aligned}$$

If $f_1(M)$ is convex (or concave) and $f_2(M)$ is monotonic, then $f_1(M)$ is fitted using C-splines and $f_2(M)$ is fitted using I-splines, and the model (1) is given by

6 $$\begin{aligned} \begin{aligned} Y&= [1, A, CS \bullet A, IS \bullet (1 - A), C] [\beta _0, \beta _1, \beta _{2}, \beta _{3}, \beta _4]^T + \epsilon _1. \end{aligned} \end{aligned}$$

### Statistical Inference

Regression parameters of the model (1) are estimated along the lines of Meyer (2018), but the method is modified to account for factor-by-curve interaction.

Define $CS = [CS_1, CS_2]$, where $CS_1 = M$ and $CS_2 = [C_1(M|2, t), ..., C_k$ (*M*|2, *t*)]. For model (3), $W_0 = [1], W = [A, C], Z_1 = [IS \bullet A], Z_0 = [IS \bullet (1 - A)] \text { and } Z = [Z_1, Z_0]$; for model (4), $W_0 = [1, CS_1 \bullet A, CS_1 \bullet (1 - A)], W = [A, C], Z_1 = [CS_2 \bullet A], Z_0 = [CS_2 \bullet (1 - A)] \text { and } Z = [Z_1, Z_0]$; for model (5), $W_0 = [1, CS_1 \bullet (1 - A)], W = [A, C], Z_1 = [IS \bullet A], Z_0 = [CS_2 \bullet (1 - A)] \text { and } Z = [Z_1, Z_0]$; for model (6), $W_0 = [1, CS_1 \bullet A], W = [A, C], Z_1 = [CS_2 \bullet A], Z_0 = [IS \bullet (1 - A)] \text { and } Z = [Z_1, Z_0]$.

Let $V = [W_0, W]$, $P_V = V (V^T V)^{-1} V^T$, the orthogonal projection operator onto the column space of $V^T$, and $\Delta = (I - P_V)Z$. Using the hinge algorithm for cone projection (Meyer 2013), a subset of columns of $\Delta$ are determined. We then keep the corresponding columns of *Z* and estimate the parameters of the model (1) using the ordinary least squares. The parameters corresponding to the eliminated columns of *Z* are estimated as 0. During the process of the hinge algorithm, if the signs of coefficients for the exposure or non-exposure group splines are assumed to be non-positive, i.e., the curve is assumed to be decreasing or concave, then we will use $-IS$ or $-CS_2$ instead of *IS* or $CS_2$. We estimate parameters of the model (2) using ordinary least squares.

Under the assumptions described in Section 2.1, we parametrize the expected controlled direct effect, natural direct effect, and natural indirect effect as follows in Proposition 1. The detailed derivation is provided in the supplementary text.

#### Proposition 1

Suppose (1) for an individual with actual exposure $A = a$, the actual outcome *Y* is $Y_a$ (consistency), (2) $Y_{am} \perp A | C$, (3) $Y_{am} \perp M | {A, C}$, (4) $M_a \perp A | C$, and (5) $Y_{am} \perp M_{a^*} | C$, and suppose models (1) and (2) are correctly specified, then the expected controlled direct effect, natural direct effect, and natural indirect effect, conditional on $C \!=\! c$, are given by

7 $$\begin{aligned} E[Y_{am} - Y_{a^*m}|c] = (\beta _1 + f_1(m) - f_2(m))(a - a^*), \end{aligned}$$

8 $$\begin{aligned} E[Y_{aM_{a^*}}-Y_{a^*M_{a^*}}|c] = (\beta _1 + E[f_1(M)|a^*, c] - E[f_2(M)|a^*, c])(a - a^*), \end{aligned}$$

and

9 $$\begin{aligned} E[Y_{aM_a}-Y_{aM_{a^*}}|c]= & {} a(E[f_1(M)|a, c] - E[f_1(M)|a^*, c]) \nonumber \\{} & {} + (1 - a)(E[f_2(M)|a, c] - E[f_2(M)|a^*, c]), \end{aligned}$$

respectively.

According to Proposition 1, the expected CDE is a function of $\beta _1$, $\beta _2$ and $\beta _3$, the expected NDE is a function of $\beta _1, \beta _2$, $\beta _3$, $\gamma _0$, $\gamma _1$, $\gamma _2$ and $\sigma _2^2$, and the expected NIE is a function of $\beta _2$, $\gamma _0$, $\gamma _1$, $\gamma _2$ and $\sigma _2^2$. Thus, these mediation effects are derived by plugging in the suitable least squares or constrained estimators. We apply the delta method to obtain the asymptotic variances of different mediation effects. All unknown parameters in the asymptotic variance expressions are replaced by the point estimates obtained from the least squares. The 95% confidence intervals are derived using the standard formula $(g(\hat{\theta }) - z_{0.975} \sqrt{\widehat{var(g(\hat{\theta }))}}, g(\hat{\theta }) + z_{0.975} \sqrt{\widehat{var(g(\hat{\theta }))}})$. The technical details are provided in Proposition 3 in the supplementary text, and the computational details are also provided in the supplementary text.

## Simulation Study

We evaluate the performance of our methodology in terms of the coverage probability (or the probability that the 95% confidence interval contains the true mediation effect), average absolute relative bias, and average mean squared error (MSE). Data were simulated to resemble the state-wide prenatal screening program data presented in the Application section (Section 4). The simulation study is intended to be illustrative of the methodology. In reality, the situation can be more complex. The simulated data set contains 500 observations and 10 variables. The confounding variables are age (randomly sampled from 18 to 40 years with an increment 0.5 years), inverse maternal weight (randomly sampled from 0.0020 to 0.0143 lbs$^{-1} \times 10^3$ with an increment of 0.0001 lbs$^{-1} \times 10^3$), race (randomly sampled from race 1 to race 5 with probabilities 0.46, 0.28, 0.13, 0.1 and 0.03 respectively), season of blood draw (randomly sampled from season 1 to season 4 with the same probability 0.25), smoking status (randomly sampled from a binomial distribution with 5% chance of smoking), ovum donor status (randomly sampled from a binomial distribution with 2% chance of donation), and pre-existing diabetes status (randomly sampled from a binomial distribution with 5% chance of diabetes). The exposure variable is pesticide exposure (randomly sampled from a binomial distribution with 50% chance of being exposed). The mediator variable is hormone (gestational age multiple of median, calculated via exposure-mediator model). The outcome variable is birth weight (grams, calculated via exposure-outcome model).

**Fig. 2:**
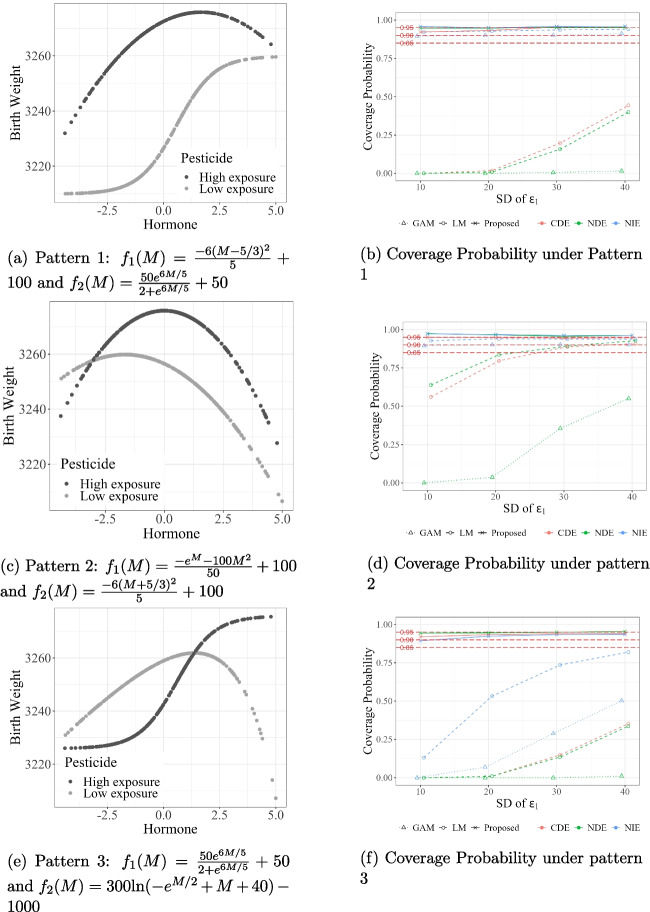
Plots of hormone vs. birth weight under each pattern, and simulation results of coverage probability for each pattern

We consider 3 different combinations of nonlinear functions for exposure-outcome model, which are shown in Fig. 2a, c and e. We fix the variance of $\epsilon _2$ in exposure-mediator model (model (2)) at $0.3^2$, and four different patterns of variances are considered for $\epsilon _1$ in exposure-outcome model (model (1)), namely, $N(0, 10^2)$, $N(0, 20^2)$, $N(0, 30^2)$, and $N(0, 40^2)$. The number of bases is set to 5. For the CDE, the mediator is set to its mean value. Since in simulation studies all parameters such as $\beta _1$, $\gamma _0$, $\gamma _1$, $\gamma _2$, and $\sigma _2^2$ and the functions $f_1(m)$ and $f_2(m)$ are known, the true effects can be calculated using the formulas in Proposition 3 to investigate the performance of the proposed methodology. The number of simulations considered is 500. The results of coverage probability are shown in Fig. 2b, d and f (“LM” in the legend of each figure corresponds to the linear regression-based method and “GAM” in the legend of each figure corresponds to the GAM (simulation)-based method), and the results of average absolute relative bias and average MSE are shown in Fig. S.1 in the supplementary text (also see Tables S.1 - S.3 in the supplementary text for details).

Under Pattern 1 (Fig. 2a), the relationship between hormone and birth weight in the high exposure group is concave (with increasing trend), and the relationship in the low exposure group is increasing (sigmoid). The true CDE is centered at 44.62 (SD = 0.14), the true NDE is 45.82, and the true NIE is 1.10. The coverage probabilities of CDE, NDE, and NIE remain at around 95% for the proposed method under different $\sigma _1$’s, but the coverage probabilities of CDE and NDE are low for the linear regression-based method, and the coverage probabilities of NDE are low for the GAM (simulation)-based method, especially in the cases of small $\sigma _1$’s. The average absolute relative biases and the average MSEs for the proposed method are much lower than those for the linear regression-based or GAM (simulation)-based method. Under Pattern 2 (Fig. 2c), the relationship between hormone and birth weight in the high exposure group is concave, and the relationship in the low exposure group is concave (with decreasing trend). The true CDE is centered at 19.85 (SD = 0.05), the true NDE is 19.17, and the true NIE is -0.17. The coverage probabilities of CDE, NDE, and NIE are approximately 95% for the proposed method. Although the coverage probabilities of the CDE and NDE for the linear regression-based method and the coverage probabilities of NDE for the GAM (simulation)-based method under Pattern 2 performed better than those under Pattern 1, they are still low in the cases of small $\sigma _1$’s. The average absolute relative biases for all methods converged as $\sigma _1$ increases. The average MSEs of CDE and NDE for the linear regression-based method and the average MSEs of NDE for the GAM (simulation)-based method are lower under large $\sigma _1$’s. The reason could be that the estimates of effects do not deviate much from the truth for the linear regression-based or GAM (simulation)-based method. Under Pattern 3 (Fig. 2e), the relationship between hormone and birth weight in the high exposure group is increasing (sigmoid), and the relationship in the low exposure group is concave. The true CDE is centered at -15.00 (SD = 0.14), the true NDE is -16.24, and the true NIE is 4.08. The coverage probabilities of CDE, NDE, and NIE remain at approximately 95% for the proposed method under different $\sigma _1$’s, but the coverage probabilities of CDE, NDE, and NIE are low for the linear regression-based method, and the coverage probabilities of NDE and NIE are low for the GAM (simulation)-based method, especially in the cases of small $\sigma _1$’s. The average absolute relative biases and the average MSEs for the proposed method are notably lower than those for the linear regression-based method. The true NIE under Pattern 3 is larger than the true NIEs under other patterns in our simulation study. For this reason, the performance measures of NIE for the linear regression-based or GAM (simulation)-based method are worse.

In summary, if $f_1(M)$ and $f_2(M)$ deviate from linear shapes, the semi-parametric shape-restricted regression spline outperforms the linear regression or GAM (simulation) in general. Using the semi-parametric shape-restricted regression spline, the coverage probability stays constant at around 95%; whereas using the linear regression or GAM (simulation), the coverage probability tends to be 0 when the variance of $\epsilon _1$ is small, and the effect size is large (see Proposition 2). Both average absolute relative bias and average MSE of the estimated effects from the semi-parametric shape-restricted regression spline increase when the variance of $\epsilon _1$ increases, but they are still smaller compared to the linear regression or GAM (simulation), especially for small variance of $\epsilon _1$ and large effect size.

For a fair comparison, we have also assumed the true relationships between hormone and birth weight in high and low exposure groups as linear, i.e., $f_1(M)=5.5M + 70$ and $f_2(M)=9.5M + 60$, both increasing (see Fig. 5a in the supplementary text). The true CDE is centered at 25.44 (SD = 0.05), the true NDE is 26.07, and the true NIE is 1.65. Because the underlying relationships are linear, the linear regression-based method performs better, as expected. The metrics are quite comparable to those from the semi-parametric shape-restricted regression spline (see Fig. S.2 and Table S.4 in the supplementary text).

### Proposition 2

If the mediation effect based on the linear regression model is different from the true mediation effect, then as the variance of $\epsilon _1$ $\rightarrow 0$, the coverage probability (or the probability that the confidence interval contains the true mediation effect) $\rightarrow 0$.

The proof of Proposition 2 can be found in the supplementary text.

## Application

Pesticides are used widely in agriculture to control pests and improve yields. By design, they are toxic to insects, and therefore we and others hypothesize unintended negative consequences on human health (Larsen et al. 2017). Larsen et al. (2017) reported that for individuals in a high pesticide exposure group (defined by pounds of pesticides applied in residential vicinity) in $1^{st}$-trimester pregnancies, birth weight was 13 grams lower. Being in the high exposure group was associated with lower gestational age, higher risk of preterm birth, and higher probability of a birth abnormality. Chemicals may not directly reach and/or affect the fetuses but instead they alter the levels of placental biomarkers, which influence the birth outcomes. This is a placentally-mediated effect (Adibi et al. 2021a). It is also possible that the environmental chemical is associated with the outcome by way of a nonlinear relationship. Once in the body, some chemicals mimic naturally occurring hormones, prostaglandins, and/or growth factors (le Maire et al. 2010). To evaluate this, we performed mediation analysis using the semi-parametric shape-restricted regression analysis developed in this paper.

**Fig. 3:**
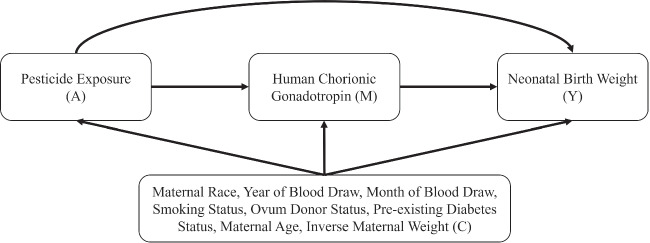
The directed acyclic graph (DAG) for the analysis

Pesticide data were publicly available through the California’s Pesticide Use Reporting (PUR) program, where the pesticide application is reported daily. The data were mapped to the zip code of maternal residence, and to the month and year of blood draw. Each woman was assigned pesticide exposure values in log pounds of pesticides, applied in that zip code during the month in which she had her blood drawn in the first trimester of pregnancy (10-14 weeks gestation). These data were merged with the prenatal screening data in which each woman had precise and accurate measures of placental-fetal hormones. Human chorionic gonadotropin (hCG) was selected as a mediator for this analysis as it is one of five placental-fetal biomarkers that is used widely to screen for fetal aneuploidy and therefore is available in the medical record for research (Malone et al. 2005). hCG is a placental glycoprotein that has been related to almost all placental functions including the transfer of nutrition and, fetal growth and development (Filicori et al. 2005; Licht et al. 2007). hCG has also been widely associated with environmental exposures including pesticides (Paulesu et al. 2018; Adibi et al. 2021b). Furthermore, Barjaktarovic et al. (2017) demonstrated in Generation R that hCG in the late first trimester was associated with birth weight in a sex-specific and nonlinear pattern. The pesticide application data was dichotomized at the median to represent low and high exposure categories (*A*). We focused on two commonly used pesticides, namely, permethrin and glyphosate isopropylamine salt. The mediator (*M*) was her serum level human chorionic gonadotropin (hCG), normalized for gestational day of blood draw, which is called the gestational age multiple of the median (GA-MoM) (Adibi et al. 2015). The outcome variable (*Y*) was neonatal birth weight, which was abstracted from the medical record for each baby. Analyses were stratified by baby sex due to previous reports on a sex difference in the hCG-birth weight association (Barjaktarovic et al. 2017). All models were adjusted for a small set of confounders of exposure-outcome, exposure-mediator, and mediator-outcome that included maternal race, year of blood draw, month of blood draw, smoking status, ovum donor status, pre-existing diabetes status, maternal age, and inverse maternal weight (Adibi et al. 2015). Information on confounders was limited to a one-page questionnaire which is completed by subjects at the time of blood draw. The DAG is shown in Fig. 3.

For the organophosphate pesticide permethrin ($N_{male} \!=\! 21,433, N_{female}$ $= 20,895$), the association between first trimester hCG and neonatal male birth weight was increasing in the above-median exposure group and concave with an increasing trend in the below-median exposure group. The relationship between first trimester hCG and neonatal female birth weight was concave with an increasing trend in the above-median exposure group and increasing in the below-median exposure group. For glyphosate isopropylamine salt ($N_{male} = 56,299, N_{female} = 55,052$), the relationship between first trimester hCG and infant male birth weight was concave with an increasing trend in the above-median exposure group and increasing in the below-median exposure group. The relationship between first trimester hCG and infant female birth weight was increasing in the above-median exposure group and concave with increasing trend in the below-median exposure group. Mediation analysis was performed using the semi-parametric shape-restricted regression spline. The number of bases was set to 5 as in the simulation study, and the confounding variables were controlled at their mean values. For the CDE, the mediator was set to its mean value. This is intended to represent a hypothetical intervention whereby we would block the placental hCG mechanism by which the pesticides affect birthweight. The results are summarized in Table 1.

**Table 1:** Mediation Analysis Results on State-Wide Prenatal Screening Program Data

Female Infant			
	CDE with 95% C.I.	NDE with 95% C.I.	NIE with 95% C.I.
Permethrin	13.270 (-4.0927, 30.633)	6.7042 (-5.2455, 18.654)	-1.1889 (-2.0636, -0.3142)
Glyphosate isopropylamine salt	4.9524 (-6.9646, 16.869)	8.8868 (-11.683, 29.457)	-1.6303 (-2.2427, -1.0178)
Male Infant			
	CDE with 95% C.I.	NDE with 95% C.I.	NIE with 95% C.I.
Permethrin	12.454 (-3.5468, 28.455)	15.665 (3.5325, 27.797)	-2.4189 (-3.5479, -1.2899)
Glyphosate isopropylamine salt	8.0234 (-3.0360, 19.083)	5.8625 (-1.6837, 13.409)	-2.4338 (-3.1993, -1.6683)

According to the results summarized in Table 1, the NIE is statistically significant at 5% level of significance in each case, as the 95% confidence intervals do not include the null value, indicating that first trimester hCG was in an association pathway between permethrin and glyphosate isopropylamine salt exposure and birth weight within male and female infants in this cohort. In our analysis, we discovered positive associations between permethrin and birth weight and between glyphosate isopropylamine salt and birth weight. After accounting for mediation, these associations became negative. It is important to note that pesticide exposure is based on application of pesticides and geographic proximity. Results can be interpreted at the aggregate level of women living in geographic proximity to specific levels of pesticide application in early pregnancy. At the individual level, misclassification of internal pesticide dose is likely.

## Discussion

The field of causal inference (and mediation analysis) continues to evolve and expand into novel directions such as high dimensional genomic data (Sohn and Li 2019; Wu et al. 2022), and others reviewed in Qin (2024). In many such instances, it is not reasonable to assume linear relationships among variables. Hence, as done in this paper, there is a need to apply nonparametric/semiparametric approaches. The methodology presented in this paper is specifically addressing the mathematical relationship between the mediator and the outcome. We are not introducing assumptions beyond those already specified in the causal mediation methodological literature. The methodology solves a problem in the setting of causal mediation analysis where there is a known or suspected nonlinear relationship between the mediator and the outcome. This method is designed to relax the linearity assumption and reduce bias introduced by model misspecification. The exposure-outcome model is specified using the quadratic I-spline basis functions and/or cubic C-spline basis functions. This allows the investigator to apply pre-existing knowledge on the underlying nonlinear relationship to their analysis. Once the shapes of the associations are established, the mediation effects can be estimated and inferred.

We demonstrate the proposed method in an applied example in environmental health and fetal origins epidemiology. In the case of placental-fetal biomarkers and fetal growth, nonlinearity is a reasonable assumption. We found that the shapes of the placental biomarker-birth weight relationships differed by the low and high pesticide application groups. One interpretation is that the shape of association in the low exposure group more closely represents the “normal” developmental relationship, and the changing of that shape in the high exposure group could indicate a type of toxicity. This information was accounted for in the estimation of the controlled direct effect (the exposure effect when hypothetically blocking the effect of the mediator), the natural direct effect (the exposure effect when assuming the mediator was unaffected by exposure), and the natural indirect effect (the effect of exposure assuming mediation). In this case, it allowed for comparisons of effect magnitude, direction, and precision of the effect across different pesticides and hormones, stratified by sex of the infant.

As shown in the Application section (Section 4), the estimation of the natural indirect effect changed the interpretation of the exposure effect. Based on the natural direct effect alone, the conclusion was that the pesticide application was either not or positively associated with the birth weight. However, based on the natural indirect effect and the consideration of the shapes, the conclusion was that the increased pesticide application would lead to the lower birth weight. This question as to whether the true exposure effect is positive or negative in direction can be further explored in studies where exposures are measured in individual pregnancies by pesticide biomarkers. A possible explanation is that the placental mechanism of toxicity differs from the direct effect, and both directions of association (positive natural direct effect and negative natural indirect effect) may be accurate. It is also important to note that, if the relationships are modeled using standard linear models, then from equations $E[Y_{aM_{a^*}}-Y_{a^*M_{a^*}}|c] = (\beta _1 + \beta _3 (\gamma _0 + \gamma _1 a^* + \gamma _2 c))(a - a^*)$ and $E[Y_{aM_a}-Y_{aM_{a^*}}|c] = (\beta _2 \gamma _1 + \beta _3 \gamma _1 a)(a - a^*)$ (VanderWeele 2015), we see that under some conditions on the model parameters, $E(NDE)> 0 > E(NIE)$. This can happen, for example, when in the exposure-outcome model, the exposure, the mediator, as well as the interaction between the exposure and the mediator, are positively correlated with the outcome. Conversely, in the exposure-mediator model, the confounders and the mediator are positively correlated while the exposure and the mediator are negatively correlated. There are potentially other configurations leading to $E(NDE)> 0 > E(NIE)$. Thus, it should not be surprising if in practice, whether we use the linear or the proposed shape-restricted regression, the natural direct effect and the natural indirect effect have opposite signs as seen in our example.

Using these estimation methods in the causal inference framework requires consideration of specific sources of bias and rigorous validation of the necessary assumptions including no unmeasured confounding, consistency, positivity, and no interference. Correct model specification is a required assumption in this framework, which is addressed in this method. Detailed discussion of these assumptions and approaches to validate them are outside the scope of this paper and are described in detail elsewhere (Hernan and Robins 2020; Nguyen et al. 2021).

The proposed method performed as desired in terms of coverage probability, average absolute relative bias, and average mean squared error based on the simulation results. This was true especially when the underlying shape was nonlinear with a specific shape being identified. Standard linear or polynomial regressions are rigid in shapes, so the corresponding 95% confidence intervals barely covered the true effect. Because the proposed method is developed using the asymptotic properties of regression analysis, it is not computationally intensive. The proposed method is a compromise between the standard linear or polynomial regressions and GAMs because it does not require a specific functional relationship nor does it allow arbitrary relationships. Although the fits are robust to the choice of smoothing parameters when assumptions about both shape and smoothness are warranted (Meyer 2008), in order to make more precise predictions, the number of knots and the knots placement may still need to be considered. See for example, Jelsema et al. (2019). The proposed method is not suitable if shapes other than monotonic, convex, and concave are present. In such cases, researchers can apply the simulation-based method developed by Imai et al. (2010). The proposed method only accommodates a categorical exposure variable. This is a limitation to be addressed in the future extension of this approach.

## Supplementary Information

Below is the link to the electronic supplementary material.

Supplementary file 1 (pdf 3793 KB)
