# Supplementary material for: Mediation Analysis using Semi-parametric Shape-Restricted Regression with Applications
Source: Sankhya Ser B. 2024 Jul 2;86(2):669–89. doi: 10.1007/s13571-024-00336-w (PMC11615969; doi:10.1007/s13571-024-00336-w)
Supplement: Supplementary file 1 — (pdf 3793 KB) [file 13571_2024_336_MOESM1_ESM.pdf]

Supplementary Text

S.1. M-splines, I-splines and C-splines

Let $t = \{t_1, t_2, ..., t_{n+k}\}$ denote the knot sequence, n denote the number of free parameters that specify the spline function having the specified continuity characteristics, and k be the order of the basis functions. Then the recursive form of M-splines is as follows (Ramsay, 1988):

For order $k = 1$, $M_i(x|1,t) = \frac{1}{t_{i+1}-t_i}$ if $t_i \leq x < t_{i+1}$, otherwise $M_i(x|1,t) = 0$, and
for order $k > 1$, $M_i(x|k,t) = \frac{k[(x-t_i)M_i(x|k-1,t)+(t_{i+k}-x)M_{i+1}(x|k-1,t)]}{(k-1)(t_{i+k}-t_i)}$ if $t_i \leq x < t_{i+k}$, otherwise $M_i(x|k,t) = 0$.

The quadratic I-splines $I_i(x|2,t)$ are obtained by integrating the M-splines of degree 1 and can be expressed as:

$I_i(x|2,t) = 0$ if $x < t_i$,
$I_i(x|2,t) = \frac{(x-t_i)^2}{(t_{i+2}-t_i)(t_{i+1}-t_i)}$ if $t_i \leq x < t_{i+1}$,
$I_i(x|2,t) = 1 - \frac{(t_{i+2}-x)^2}{(t_{i+2}-t_i)(t_{i+2}-t_{i+1})}$ if $t_{i+1} \leq x < t_{i+2}$, and
$I_i(x|2,t) = 1$ if $x \geq t_{i+2}$.

The cubic C-splines $C_i(x|2,t)$ are obtained by integrating the quadratic I-splines and can be expressed as:

$C_i(x|2,t) = 0$ if $x < t_i$,
$C_i(x|2,t) = \frac{(x-t_i)^3}{3(t_{i+2}-t_i)(t_{i+1}-t_i)}$ if $t_i \leq x < t_{i+1}$,
$C_i(x|2,t) = x - \frac{t_i+t_{i+1}+t_{i+2}}{3} + \frac{(t_{i+2}-x)^3}{3(t_{i+2}-t_i)(t_{i+2}-t_{i+1})}$ if $t_{i+1} \leq x < t_{i+2}$, and
$C_i(x|2,t) = x - \frac{t_i+t_{i+1}+t_{i+2}}{3}$ if $x \geq t_{i+2}$.

S.2. Proof of Proposition 1

Proof. Under the conditions described in Proposition 1, and with the models (1) and (2) being correctly specified, we obtain after some simplifications:

$E[CDE|c] = E[Y_{am} - Y_{a^*m}|c] = E[Y|a,m,c] - E[Y|a^*,m,c] = (\beta_1 + f_1(m) - f_2(m))(a - a^*)$,

$E[NDE|c] = E[Y_{aM_{a^*}} - Y_{a^*M_{a^*}}|c] = \int_m \{E[Y|a,m,c] - E[Y|a^*,m,c]\} f(m|a^*,c)dm$
$= (\beta_1 + E[f_1(M)|a^*,c] - E[f_2(M)|a^*,c])(a - a^*)$, and
$E[NIE|c] = E[Y_{aM_a} - Y_{aM_{a^*}}|c] = \int_m E[Y|a,m,c]\{f(m|a,c) - f(m|a^*,c)\}dm$
$= a(E[f_1(M)|a,c] - E[f_1(M)|a^*,c]) + (1-a)(E[f_2(M)|a,c] - E[f_2(M)|a^*,c])$.

Let the knot sequence be $L = t_1 = t_2 < t_3 < ... < t_k < t_{k+1} = t_{k+2} = U$. If $f_1(M)$ is fitted using I-splines, then $f_1(M) = \beta_{21}I_1(M|2,t) + ... + \beta_{2k}I_k(M|2,t)$. This is a piece-wise function, where, for $t_k \le M < t_{k+1}$, $f_1(M) = \beta_{21} + ... + \beta_{2,k-2} + \beta_{2,k-1}(1 - \frac{(t_{k+1}-M)^2}{(t_{k+1}-t_k)(t_{k+1}-t_{k-1})}) + \beta_{2,k}(\frac{(M-t_k)^2}{(t_{k+1}-t_k)(t_{k+2}-t_k)})$. Then conditional on a and c we obtain:

$E[f_1(M)|a,c] = \int_m (\beta_{21}I_1(m|2,t) + ... + \beta_{2k}I_k(m|2,t))f(m|a,c)dm = \sum_{i=2}^{k}\{\int_{t_i}^{t_{i+1}}[\beta_{21} + ... + \beta_{2,i-2} + \beta_{2,i-1}(1 - \frac{(t_{i+1}-m)^2}{(t_{i+1}-t_i)(t_{i+1}-t_{i-1})}) + \beta_{2,i}(\frac{(m-t_i)^2}{(t_{i+1}-t_i)(t_{i+2}-t_i)})]f(m|a,c)dm\}$, where $f(m|a,c)$ denotes normal density with mean $\gamma_0 + \gamma_1 a + \gamma_2 c$ and variance σ_2^2. Similar expressions can be derived for $f_2(M)$ and $E[f_2(M)|a,c]$.

If $f_1(M)$ is fitted using C-splines, then $f_1(M) = \beta_{20}M + \beta_{21}C_1(M|2,t) + ... + \beta_{2k}C_k(M|2,t)$, which is also a piece-wise function. For either $M < t_2$ or $M \ge t_{k+1}$, $f_1(M) = \beta_{20}M$. For $t_k \le M < t_{k+1}$, $f_1(M) = \beta_{20}M + \beta_{21}(M - \frac{t_1+t_2+t_3}{3}) + ... + \beta_{2,k-2}(M - \frac{t_{k-2}+t_{k-1}+t_k}{3}) + \beta_{2,k-1}(m - \frac{t_{k-1}+t_k+t_{k+1}}{3} + \frac{(t_{k+1}-m)^3}{3(t_{k+1}-t_k)(t_{k+1}-t_{k-1})}) + \beta_{2,k}(\frac{(m-t_k)^3}{3(t_{k+1}-t_k)(t_{k+2}-t_k)})$. Then conditional on a and c we obtain:

$E[f_1(M)|a,c] = \int_m (\beta_{20}m + \beta_{21}C_1(m|2,t) + ... + \beta_{2k}C_k(m|2,t))f(m|a,c)dm =$
$\beta_{20}(\gamma_0 + \gamma_1 a + \gamma_2 c) + \sum_{i=2}^{k}\{\int_{t_i}^{t_{i+1}}[\beta_{21}(m - \frac{t_1+t_2+t_3}{3}) + ... + \beta_{2,i-2}(m - \frac{t_{i-2}+t_{i-1}+t_i}{3})$
$+\beta_{2,i-1}(m - \frac{t_{i-1}+t_i+t_{i+1}}{3} + \frac{(t_{i+1}-m)^3}{3(t_{i+1}-t_i)(t_{i+1}-t_{i-1})}) + \beta_{2,i}(\frac{(m-t_i)^3}{3(t_{i+1}-t_i)(t_{i+2}-t_i)})]f(m|a,c)dm\}$.

Similar expressions can be derived for $f_2(M)$ and $E[f_2(M)|a,c]$. □

S.3. Proposition 3

Proposition 3. *Let $\theta_{CDE} = (\beta_1, \beta_2, \beta_3)$, $\theta_{NDE} = (\beta_1, \beta_2, \beta_3, \gamma_0, \gamma_1, \gamma_2, \sigma_2^2)$ and $\theta_{NIE} = (\beta_2, \gamma_0, \gamma_1, \gamma_2, \sigma_2^2)$, where $\beta_2 = (\beta_{21}, ..., \beta_{2k})$ and $\beta_3 = (\beta_{31}, ..., \beta_{3k})$ in case of quadratic I-splines, and $\beta_2 = (\beta_{20}, \beta_{21}, ..., \beta_{2k})$ and $\beta_3 = (\beta_{30}, \beta_{31}, ..., \beta_{3k})$ in case of cubic C-splines.*

Denote the expected controlled direct effect as $g_{CDE}(\theta_{CDE})$, the expected natural direct effect as $g_{NDE}(\theta_{NDE})$ and the expected natural indirect effect as $g_{NIE}(\theta_{NIE})$. Then the asymptotic variances of expected CDE, NDE and NIE are $\nabla_{\theta_{CDE}} g_{CDE}(\theta_{CDE})^T \Sigma_{\theta_{CDE}} \nabla_{\theta_{CDE}} g_{CDE}(\theta_{CDE})$, $\nabla_{\theta_{NDE}} g_{NDE}(\theta_{NDE})^T \Sigma_{\theta_{NDE}} \nabla_{\theta_{NDE}} g_{NDE}(\theta_{NDE})$ and $\nabla_{\theta_{NIE}} g_{NIE}(\theta_{NIE})^T \Sigma_{\theta_{NIE}} \nabla_{\theta_{NIE}} g_{NIE}(\theta_{NIE})$ respectively, where $\Sigma_{\theta_{CDE}}$, $\Sigma_{\theta_{NDE}}$ and $\Sigma_{\theta_{NIE}}$ are the covariance matrices corresponding to the estimated θ_{CDE}, θ_{NDE} and θ_{NIE}.

If $f_1(M)$ is fitted using I-splines, then:

$\frac{\partial E[f_1(M)|a,c]}{\partial \beta_{2i}} = \int_{t_i}^{t_{i+1}} \frac{(m-t_i)^2}{(t_{i+1}-t_i)(t_{i+2}-t_i)} f(m|a,c)dm + \int_{t_{i+1}}^{t_{i+2}} (1 - \frac{(t_{i+2}-m)^2}{(t_{i+2}-t_{i+1})(t_{i+2}-t_i)}) f(m|a,c)dm + \int_{t_{i+2}}^{t_{i+3}} f(m|a,c)dm + ... + \int_{t_k}^{t_{k+1}} f(m|a,c)dm$, for $i = 1, 2, \ldots, k$,

$\frac{\partial E[f_1(M)|a,c]}{\partial \gamma_0} = \sum_{i=2}^{k} \{ \int_{t_i}^{t_{i+1}} [\beta_{21} + ... + \beta_{2,i-2} + \beta_{2,i-1}(1 - \frac{(t_{i+1}-m)^2}{(t_{i+1}-t_i)(t_{i+1}-t_{i-1})}) + \beta_{2,i}(\frac{(m-t_i)^2}{(t_{i+1}-t_i)(t_{i+2}-t_i)})] f(m|a,c) \frac{2(m-(\gamma_0+\gamma_1 a+\gamma_2 c))}{2\sigma_2^2} dm \}$,

$\frac{\partial E[f_1(M)|a,c]}{\partial \sigma_2^2} = \sum_{i=2}^{k} \{ \int_{t_i}^{t_{i+1}} [\beta_{21} + ... + \beta_{2,i-2} + \beta_{2,i-1}(1 - \frac{(t_{i+1}-m)^2}{(t_{i+1}-t_i)(t_{i+1}-t_{i-1})}) + \beta_{2,i}(\frac{(m-t_i)^2}{(t_{i+1}-t_i)(t_{i+2}-t_i)})] f(m|a,c)(-\frac{1}{2\sigma_2^2} + \frac{(m-(\gamma_0+\gamma_1 a+\gamma_2 c))^2}{2(\sigma_2^2)^2}) dm \}$.

Similar expressions can be derived for $\frac{\partial E[f_1(M)|a,c]}{\partial \gamma_1}$ and $\frac{\partial E[f_1(M)|a,c]}{\partial \gamma_2}$.

If $f_1(M)$ is fitted using C-splines, then:

$\frac{\partial E[f_1(M)|a,c]}{\partial \beta_{20}} = \gamma_0 + \gamma_1 a + \gamma_2 c$,

$\frac{\partial E[f_1(M)|a,c]}{\partial \beta_{2i}} = \int_{t_i}^{t_{i+1}} \frac{(m-t_i)^3}{3(t_{i+1}-t_i)(t_{i+2}-t_i)} f(m|a,c)dm + \int_{t_{i+1}}^{t_{i+2}} (m - \frac{t_i+t_{i+1}+t_{i+2}}{3} + \frac{(t_{i+2}-m)^3}{3(t_{i+2}-t_{i+1})(t_{i+2}-t_i)}) f(m|a,c)dm + \int_{t_{i+2}}^{t_{i+3}} (m - \frac{t_i+t_{i+1}+t_{i+2}}{3}) f(m|a,c)dm + ... + \int_{t_k}^{t_{k+1}} (m - \frac{t_i+t_{i+1}+t_{i+2}}{3}) f(m|a,c)dm$, for $i = 1, 2, \ldots, k$

$\frac{\partial E[f_1(M)|a,c]}{\partial \gamma_0} = \beta_{20} + \sum_{i=2}^{k} \{ \int_{t_i}^{t_{i+1}} [\beta_{21}(m - \frac{t_1+t_2+t_3}{3}) + ... + \beta_{2,i-2}(m - \frac{t_{i-2}+t_{i-1}+t_i}{3}) + \beta_{2,i-1}(m - \frac{t_{i-1}+t_i+t_{i+1}}{3} + \frac{(t_{i+1}-m)^3}{3(t_{i+1}-t_i)(t_{i+1}-t_{i-1})}) + \beta_{2,i}(\frac{(m-t_i)^3}{3(t_{i+1}-t_i)(t_{i+2}-t_i)})] f(m|a,c) \frac{2(m-(\gamma_0+\gamma_1 a+\gamma_2 c))}{2\sigma_2^2} dm \}$,

$\frac{\partial E[f_1(M)|a,c]}{\partial \sigma_2^2} = \sum_{i=2}^{k} \{ \int_{t_i}^{t_{i+1}} [\beta_{21}(m - \frac{t_1+t_2+t_3}{3}) + ... + \beta_{2,i-2}(m - \frac{t_{i-2}+t_{i-1}+t_i}{3}) + \beta_{2,i-1}(m - \frac{t_{i-1}+t_i+t_{i+1}}{3} + \frac{(t_{i+1}-m)^3}{3(t_{i+1}-t_i)(t_{i+1}-t_{i-1})}) + \beta_{2,i}(\frac{(m-t_i)^3}{3(t_{i+1}-t_i)(t_{i+2}-t_i)})] f(m|a,c)(-\frac{1}{2\sigma_2^2} + \frac{(m-(\gamma_0+\gamma_1 a+\gamma_2 c))^2}{2(\sigma_2^2)^2}) dm \}$.

Similarly expressions can be derived for $\frac{\partial E[f_1(M)|a,c]}{\partial \gamma_1}$ and $\frac{\partial E[f_1(M)|a,c]}{\partial \gamma_2}$.

S.4. Proof of Proposition 2

Proof. Using linear regression, the exposure-outcome model is expressed as

$$Y = \beta_0 + \beta_1 A + \beta_2 M + \beta_3 AM + \beta_4 C + \epsilon_1 \tag{S.1}$$

where $\epsilon_1 \sim N(0, \sigma_1^2)$, while the exposure-mediator model keeps the same as model (2). Then the expected CDE, NDE and NIE, conditioning on $C = c$, are given by

$$E[Y_{am} - Y_{a^*m}|c] = (\beta_1 + \beta_3 m)(a - a^*), \tag{S.2}$$

$$E[Y_{aM_{a^*}} - Y_{a^*M_{a^*}}|c] = (\beta_1 + \beta_3(\gamma_0 + \gamma_1 a^* + \gamma_2 c))(a - a^*), \tag{S.3}$$

and

$$E[Y_{aM_a} - Y_{aM_{a^*}}|c] = (\beta_2\gamma_1 + \beta_3\gamma_1 a)(a - a^*), \tag{S.4}$$

respectively. Let $X = [1, A, M, AM, C]$, then $\hat{\beta} \sim N(\beta, \sigma_1^2(X^T X)^{-1})$. The expected CDE, NDE and NIE can all be expressed as a linear combination of β. Assuming $\hat{\theta}_{LR} = a\hat{\beta} \sim N(a\beta, \sigma_1^2 a(X^T X)^{-1}a^T)$, then we obtain $P(|\hat{\theta}_{LR} - \theta_{true}| \leq z_{\alpha/2}\sqrt{Var(\hat{\theta}_{LR})}) = \phi(z_{\alpha/2} + \frac{\theta_{true} - \theta_{LR}}{\sigma_1\sqrt{a(X^T X)^{-1}a^T}}) - \phi(-z_{\alpha/2} + \frac{\theta_{true} - \theta_{LR}}{\sigma_1\sqrt{a(X^T X)^{-1}a^T}})$, where $\phi(\cdot)$ denotes normal density function. Therefore, if $\theta_{true} \neq \theta_{LR}$ then as $\sigma_1 \to 0$, the coverage probability $\to 0$. □

S.5. Simulation results of average absolute relative bias and average MSE for each pattern

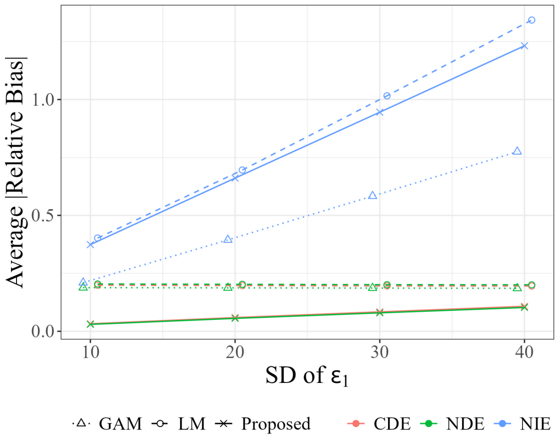

(a) Average |Relative Bias| under pattern 1

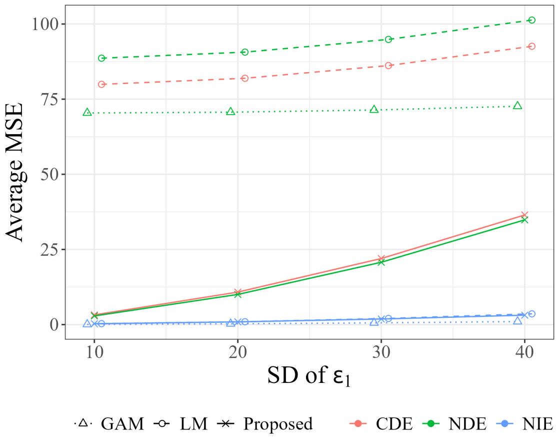

(b) Average MSE under pattern 1

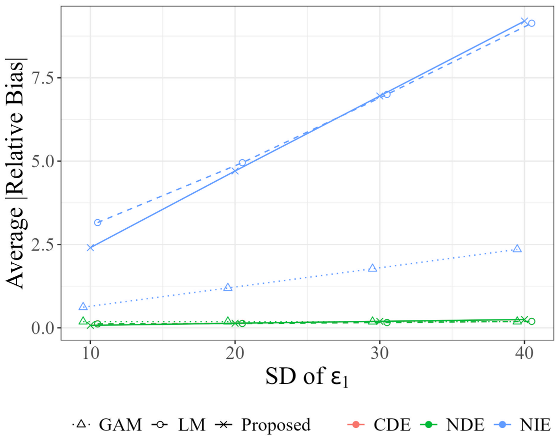

(c) Average |Relative Bias| under pattern 2

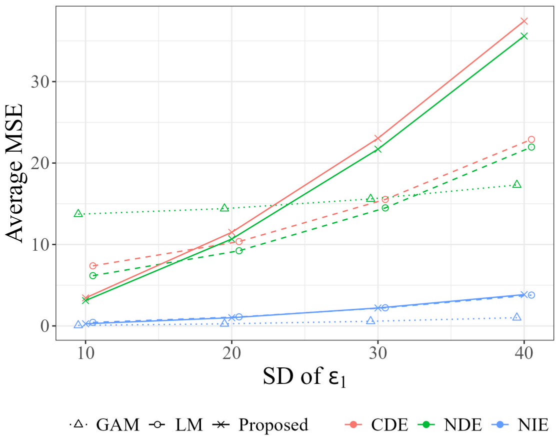

(d) Average MSE under pattern 2

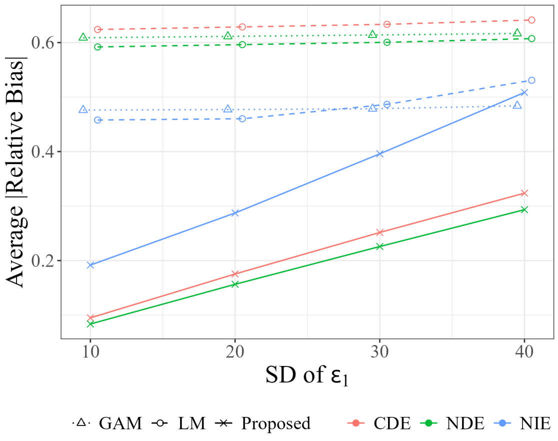

(e) Average |Relative Bias| under pattern 3

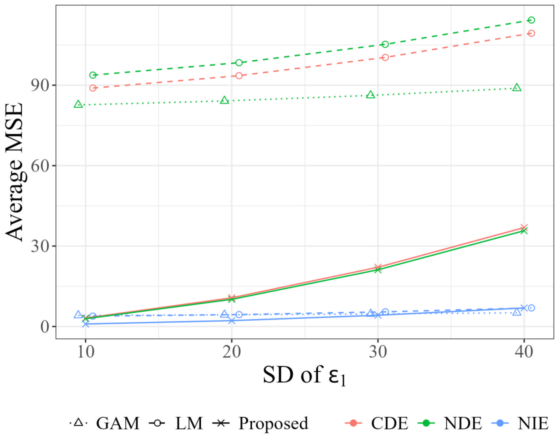

(f) Average MSE under pattern 3

Figure S.1: Simulation results of average absolute relative bias and average MSE for each pattern

S.6. Plots of hormone vs. birth weight under linear pattern and simulation results for linear pattern

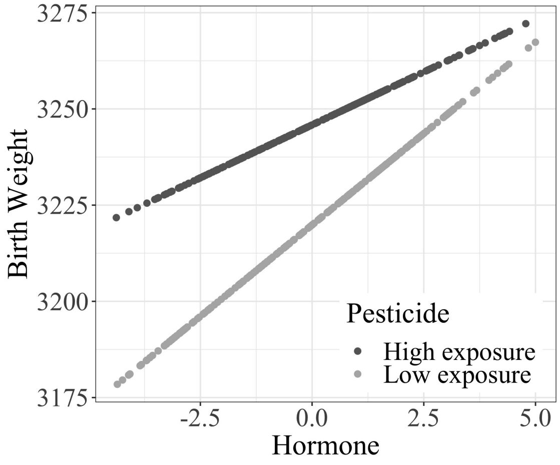

(a) Linear Pattern

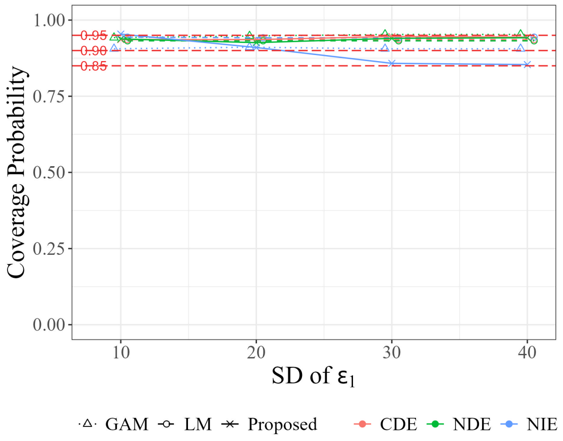

(b) Coverage Probability

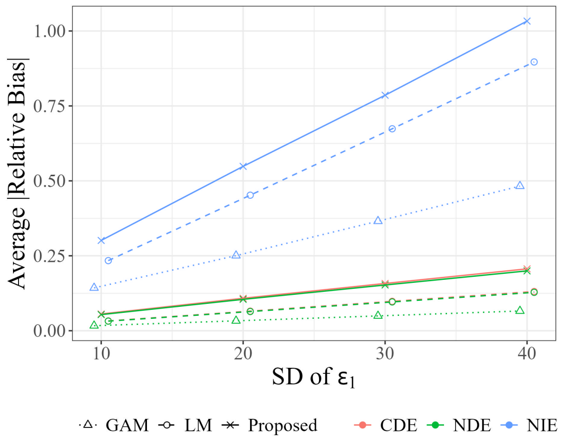

(c) Average |Relative Bias|

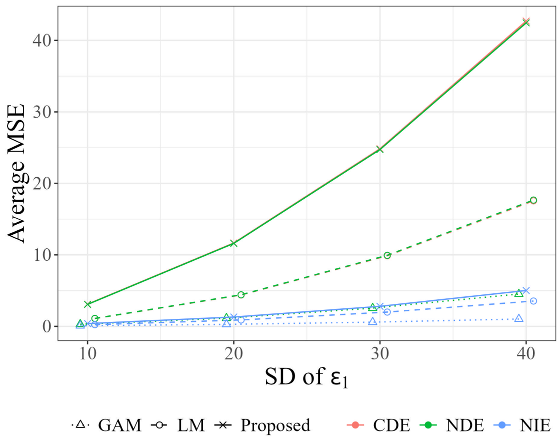

(d) Average MSE

Figure S.2: Plots of hormone vs. birth weight under linear pattern, and simulation results of coverage probability, average absolute relative bias and average MSE for linear pattern

S.7. Tables of Simulation Results

Table S.1: Simulation results of coverage probability, average absolute relative bias and average MSE for pattern 1 (true CDE: ~ 44.62, true NDE: 45.82, true NIE: 1.10)

Semi-parametric shape-restricted regression spline									
Variance of ϵ_1	Coverage Probability			Average |Relative Bias|			Average MSE		
	CDE	NDE	NIE	CDE	NDE	NIE	CDE	NDE	NIE
10^2	0.922	0.948	0.958	0.032	0.030	0.374	3.223	2.879	0.281
20^2	0.932	0.948	0.950	0.059	0.056	0.661	10.808	10.030	0.902
30^2	0.954	0.952	0.958	0.084	0.080	0.945	21.982	20.739	1.852
40^2	0.952	0.952	0.960	0.108	0.103	1.232	36.465	34.837	3.129

Linear regression									
Variance of ϵ_1	Coverage Probability			Average |Relative Bias|			Average MSE		
	CDE	NDE	NIE	CDE	NDE	NIE	CDE	NDE	NIE
10^2	0.000	0.000	0.922	0.199	0.204	0.402	79.924	88.628	0.312
20^2	0.018	0.008	0.928	0.197	0.203	0.696	81.953	90.648	0.959
30^2	0.198	0.158	0.936	0.196	0.201	1.016	86.176	94.878	2.048
40^2	0.444	0.400	0.940	0.195	0.200	1.343	92.594	101.316	3.580

Generalized additive model									
Variance of ϵ_1	Coverage Probability			Average |Relative Bias|			Average MSE		
	CDE	NDE	NIE	CDE	NDE	NIE	CDE	NDE	NIE
10^2	-	0.000	0.895	-	0.188	0.210	-	70.406	0.072
20^2	-	0.000	0.901	-	0.187	0.394	-	70.662	0.257
30^2	-	0.005	0.901	-	0.186	0.583	-	71.381	0.565
40^2	-	0.016	0.911	-	0.186	0.775	-	72.631	0.996

Table S.2: Simulation results of coverage probability, average absolute relative bias and average MSE for pattern 2 (true CDE: $\sim$ 19.85, true NDE: 19.17, true NIE: -0.17)

Semi-parametric shape-restricted regression spline									
Variance of ϵ_1	Coverage Probability			Average |Relative Bias|			Average MSE		
	CDE	NDE	NIE	CDE	NDE	NIE	CDE	NDE	NIE
10^2	0.950	0.974	0.972	0.076	0.074	2.405	3.442	3.114	0.264
20^2	0.948	0.966	0.968	0.138	0.137	4.704	11.496	10.673	1.008
30^2	0.944	0.956	0.962	0.195	0.195	6.954	23.035	21.703	2.205
40^2	0.946	0.962	0.962	0.247	0.249	9.200	37.434	35.597	3.847
Linear regression									
Variance of ϵ_1	Coverage Probability			Average |Relative Bias|			Average MSE		
	CDE	NDE	NIE	CDE	NDE	NIE	CDE	NDE	NIE
10^2	0.560	0.638	0.928	0.124	0.116	3.157	7.376	6.172	0.434
20^2	0.796	0.836	0.940	0.134	0.130	4.953	10.357	9.227	1.109
30^2	0.888	0.892	0.938	0.159	0.159	7.000	15.533	14.491	2.226
40^2	0.908	0.928	0.938	0.192	0.195	9.135	22.902	21.964	3.786
Generalized additive model									
Variance of ϵ_1	Coverage Probability			Average |Relative Bias|			Average MSE		
	CDE	NDE	NIE	CDE	NDE	NIE	CDE	NDE	NIE
10^2	-	0.000	0.895	-	0.186	0.619	-	13.722	0.068
20^2	-	0.037	0.901	-	0.185	1.195	-	14.407	0.257
30^2	-	0.356	0.901	-	0.185	1.771	-	15.593	0.571
40^2	-	0.550	0.901	-	0.187	2.351	-	17.313	1.010

Table S.3: Simulation results of coverage probability, average absolute relative bias and average MSE for pattern 3 (true CDE: $\sim$ -15.00, true NDE: -16.24, true NIE: 4.08)

Semi-parametric shape-restricted regression spline									
Variance of ϵ_1	Coverage Probability			Average |Relative Bias|			Average MSE		
	CDE	NDE	NIE	CDE	NDE	NIE	CDE	NDE	NIE
10^2	0.920	0.944	0.894	0.095	0.084	0.192	3.145	2.910	0.940
20^2	0.934	0.946	0.924	0.175	0.156	0.287	10.708	10.134	2.193
30^2	0.940	0.950	0.934	0.252	0.226	0.396	22.092	21.157	4.190
40^2	0.942	0.954	0.936	0.324	0.293	0.508	36.892	35.725	6.915
Linear regression									
Variance of ϵ_1	Coverage Probability			Average |Relative Bias|			Average MSE		
	CDE	NDE	NIE	CDE	NDE	NIE	CDE	NDE	NIE
10^2	0.000	0.000	0.130	0.624	0.592	0.458	88.930	93.747	3.865
20^2	0.010	0.010	0.532	0.629	0.596	0.460	93.545	98.385	4.448
30^2	0.148	0.134	0.736	0.634	0.601	0.487	100.355	105.232	5.473
40^2	0.352	0.336	0.820	0.641	0.607	0.531	109.360	114.288	6.940
Generalized additive model									
Variance of ϵ_1	Coverage Probability			Average |Relative Bias|			Average MSE		
	CDE	NDE	NIE	CDE	NDE	NIE	CDE	NDE	NIE
10^2	-	0.000	0.000	-	0.609	0.476	-	82.627	4.150
20^2	-	0.000	0.068	-	0.611	0.477	-	84.116	4.360
30^2	-	0.000	0.288	-	0.614	0.479	-	86.181	4.684
40^2	-	0.010	0.503	-	0.617	0.484	-	88.846	5.125

Table S.4: Simulation results of coverage probability, average absolute relative bias and average MSE for linear pattern (true CDE: $\sim$ 25.44, true NDE: 26.07, true NIE: 1.65)

Variance of ϵ_1	Coverage Probability			Average |Relative Bias|			Average MSE		
**Semi-parametric shape-restricted regression spline**									
	CDE	NDE	NIE	CDE	NDE	NIE	CDE	NDE	NIE
10^2	0.936	0.938	0.954	0.056	0.054	0.301	3.101	3.070	0.387
20^2	0.936	0.926	0.910	0.108	0.105	0.548	11.657	11.608	1.303
30^2	0.946	0.940	0.858	0.158	0.153	0.786	24.839	24.736	2.800
40^2	0.944	0.942	0.854	0.206	0.200	1.033	42.702	42.454	5.016
**Linear regression**									
Variance of ϵ_1	Coverage Probability			Average |Relative Bias|			Average MSE		
	CDE	NDE	NIE	CDE	NDE	NIE	CDE	NDE	NIE
10^2	0.936	0.932	0.944	0.033	0.032	0.234	1.097	1.107	0.239
20^2	0.936	0.932	0.942	0.065	0.064	0.452	4.389	4.417	0.898
30^2	0.936	0.932	0.942	0.098	0.096	0.674	9.875	9.936	1.999
40^2	0.936	0.932	0.944	0.131	0.128	0.897	17.556	17.664	3.543
**Generalized additive model**									
Variance of ϵ_1	Coverage Probability			Average |Relative Bias|			Average MSE		
	CDE	NDE	NIE	CDE	NDE	NIE	CDE	NDE	NIE
10^2	-	0.942	0.906	-	0.017	0.143	-	0.295	0.088
20^2	-	0.948	0.911	-	0.033	0.251	-	1.143	0.276
30^2	-	0.953	0.906	-	0.049	0.366	-	2.553	0.589
40^2	-	0.953	0.906	-	0.066	0.482	-	4.523	1.026
